# Cryptochrome-Related Abiotic Stress Responses in Plants

**DOI:** 10.3389/fpls.2018.01897

**Published:** 2018-12-19

**Authors:** Victor D’Amico-Damião, Rogério Falleiros Carvalho

**Affiliations:** Department of Biology Applied to Agriculture, São Paulo State University, São Paulo, Brazil

**Keywords:** abiotic stress acclimation, blue-light photoreceptor, cryptochromes, drought, heat, high light, salinity

## Abstract

It is well known that light is a crucial environmental factor that has a fundamental role in plant growth and development from seed germination to fruiting. For this process, plants contain versatile and multifaceted photoreceptor systems to sense variations in the light spectrum and to acclimate to a range of ambient conditions. Five main groups of photoreceptors have been found in higher plants, cryptochromes, phototropins, UVR8, zeitlupes, and phytochromes, but the last one red/far red wavelengths photoreceptor is the most characterized. Among the many responses modulated by phytochromes, these molecules play an important role in biotic and abiotic stress responses, which is one of the most active research topics in plant biology, especially their effect on agronomic traits. However, regarding the light spectrum, it is not surprising to consider that other photoreceptors are also part of the stress response modulated by light. In fact, it has become increasingly evident that cryptochromes, which mainly absorb in the blue light region, also act as key regulators of a range of plant stress responses, such as drought, salinity, heat, and high radiation. However, this information is rarely evidenced in photomorphogenetic studies. Therefore, the scope of the present review is to compile and discuss the evidence on the abiotic stress responses in plants that are modulated by cryptochromes.

## Introduction

Sunlight is the most essential environmental signal for plant growth and development. Interestingly, its influence is not restricted to the photosynthesis process because light also plays a key role in transferring essential information, such as quality, quantity, periodicity, and direction, that ensures plant survival under environmental fluctuations (Kami et al., 2010; Demarsy et al., 2018). For this reason, photomorphogenesis has been well elucidated by modulating a plethora of plant processes from seed germination to fruiting (Kendrick and Kronenberg, 1994; Kami et al., 2010). In plants, several of these photomorphogenic responses are largely governed by the visible light spectrum (400–700 nm). In fact, variations in light are sensed by photoreceptors, which are photoreversible proteins with a prosthetic group/cofactor or an intrinsic amino acid (tryptophan) as a chromophore. Currently, five photoreceptor systems have been identified in higher plants: phytochromes (phys), which absorb wavelengths of red (600–700 nm) and far-red light (700–750 nm); blue light (BL)/ultraviolet (UV)-A photoreceptors (315–500 nm), which are represented by cryptochromes (crys), phototropins (phots) and zeitlupes (ZTL, FKF1, and LKP2); and UV Resistance Locus 8 (UVR8) photoreceptor, which operate through UV-B light (280–315 nm) (Chen and Chory, 2011; Christie et al., 2012; Ito et al., 2012; Jenkins, 2014; Christie et al., 2015; Mishra and Khurana, 2017). Although there are several classes of plant photoreceptors, phytochromes were the first type of photoreceptor discovered and are the most characterized. These molecules are dimeric apoproteins (∼130 KDa) covalently linked to phytochromobilin, which is a linear tetrapyrrole that functions as a chromophore. The interconvertible forms of phytochromes allow their activation via the absorption of red light, as well as their inactivation via the absorption of far-red light (Chen and Chory, 2011). Thus, this sensor triggers the light-dependent signal transduction cascade to regulate the expression of numerous genes that result in specific physiological responses (Viczián et al., 2017). Furthermore, various reports have shown that the plant signaling pathways involved in the responses to abiotic and biotic stresses, including insect herbivory, salinity, drought, hot or cold temperatures, and UV-B radiation, are modulated by phytochromes (Donohue et al., 2008; Ballaré, 2009; Carvalho et al., 2011; D’Amico-Damião et al., 2015; Gavassi et al., 2017). However, these responses remain unclear due to the complex light signaling pathways that are operated by other photoreceptors along the light spectrum (Kami et al., 2010; Fiorucci and Fankhauser, 2017; Demarsy et al., 2018). Thus, regarding the intricate photomorphogenic organization, it is important to remember that, in addition to phytochromes, other molecules, such as crys, have also increasingly received attention with regard to light signaling.

**FIGURE 1:**
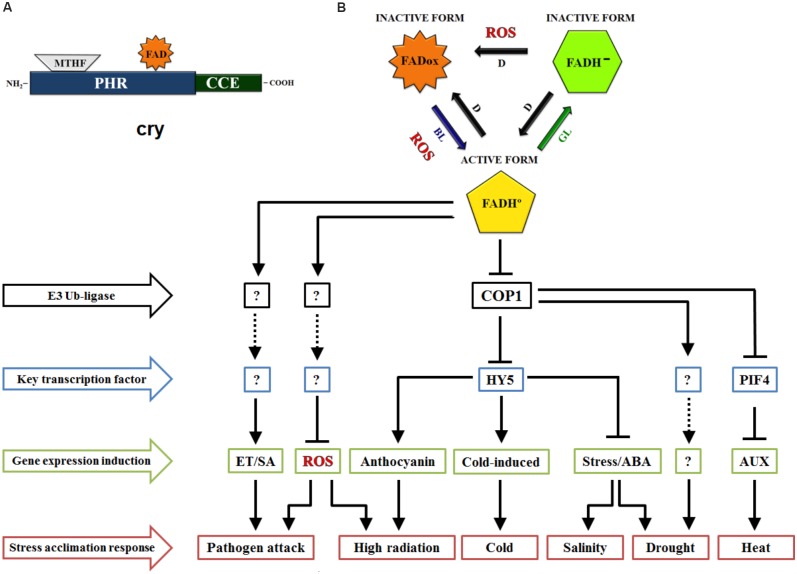
Schematic view of cryptochromes (cry) role in stress acclimation responses. **(A)** Domain structure of cry consists of Photolyase-Homologous Region (PHR) domain and CRY C-terminal extension (CCE) domain. The cry chromophores methenyltetrahydrofolate (MTHF) and FAD are binding to PHR, a light perception domain. **(B)** FAD chromophore redox states of cry. FAD is oxidized (FADox) in the darkness (D), with the absorption peak in blue light (BL). On light exposure, FADox changes to the neutral semireduced state (FADH°), which allows the biological activity of cry in the plant cell. With the absorption peak in green light (GL), FADH° is induced to change to the totally reduced form (FAD-). An unclear subject around the reoxidation of FAD is the production of reactive oxygen species (ROS) during the process. Stress signaling pathway regulated by cryptochromes. From the blue light signaling, cry coordinate the negative regulation of COP1-dependent degradation of HY5 and PIF4 transcription factors, triggering changes in stress response target genes expression. Note that these are key pathways in the induction of genes related to signaling and/or biosynthesis of hormones, ROS and stress components. Although the complex signaling of cry in stress acclimation still involves some unknown components, certainly this photoreceptor play a crucial role in BL-dependent stress responses. Arrows means downregulation, T-bars indicate upregulation and dotted lines indicate unknown (?) signaling routes. E3 ubiquitin-ligase; COP1, constitutive photomorphogenic 1; HY5, long hypocotyl 5; PIF4, phytochrome interacting factor 4; ET, ethylene; SA, salicylic acid; ABA, abscisic acid; AUX, auxin.

Increasingly, crys have been shown to modulate plant responses from germination to fruiting, especially through wavelengths of blue, green, and UV-A light (Cashmore et al., 1999; Chaves et al., 2011). Crys are present from bacteria to humans, in plants, these photoreceptors are basically an apoprotein with two prosthetic groups (Figure 1A), the N-terminal photolyase homology-related (PHR) domain and a cryptochrome C-terminal extension (CCE) domain. The PHR domain contains the non-covalent binding site for 5, 10-methenyl tetrahydrofolate (MTHF) and flavin adenine dinucleotide (FAD), the two chromophores. FAD is a chromophore for BL that triggers photomorphogenesis, and the MTHF chromophore is a derivative of pterine that acts in the UV-A region, transferring excitation energy to FAD, which is a catalytic cofactor (Mishra and Khurana, 2017). In the model plant *Arabidopsis thaliana*, three crys were identified (cry1, cry2, and cry3). Cry1 and cry2 are located predominantly in the nucleus (Cashmore et al., 1999; Kleiner et al., 1999) and play a multifaceted role in various aspects of plant growth and development. For instance, cry1 primarily regulates photomorphogenic responses related to the inhibition of hypocotyl elongation, anthocyanin accumulation and cotyledon expansion, while cry2 plays a role in the hypocotyl inhibition, circadian clock and photoperiod-dependent flowering (Yu et al., 2010). However, cry3 is a DASH protein located in chloroplasts and mitochondria (Kleine et al., 2003), which works to repair UV-damaged DNA in a light-dependent manner (Mishra and Khurana, 2017). Overall, cryptochrome-dependent signaling pathways remain unclear because these physiological responses change with BL intensity (Kami et al., 2010) as well as with the plant species in question (Yang et al., 2017).

In addition to the variety of mechanisms orchestrated by crys, the modes of action by which these extraordinary photoreceptors work are very dynamic. For example, recent studies have revealed the role of crys in BL-dependent biosynthesis of reactive oxygen species (ROS) (Consentino et al., 2015; Jourdan et al., 2015; El-Esawi et al., 2017), which is a key signaling pathway for genes, such as those related to biotic and abiotic stresses, that have ROS-dependent transcription (Suzuki and Katano, 2018). In fact, these new findings contribute to the already established knowledge regarding the acclimation responses to biotic and abiotic stress mediated by crys, such as drought, salinity, heat, freezing, high radiation, UV-B light, and pathogen attack (Mao et al., 2005; Catalá et al., 2011; Ma et al., 2016; Zhou et al., 2017, 2018; Demarsy et al., 2018). However, although this information is mixed throughout few research studies already performed in the literature, here we discuss the evidence on cryptochrome-controlled plant acclimation responses under stressful environments and how these responses may occur (Table 1). Furthermore, the present review has compiled the most classic and current advances inherent to cry defense mechanisms against abiotic stress conditions.

**Table 1:** General description of stress responses controlled by cryptochromes in plants.

Species	Type of stress	Cryptochrome	Stress response	Reference
*Arabidopsis thaliana*	Drought	cry1, cry2	COP1, a repressor of stomatal opening is downregulates by crys	Mao et al., 2005
	Drought and Salinity	cry1, cry2	Cryptochromes-induced altered expression of stress/ABA-responsive genes	Xu and Ma, 2009; Xu et al., 2009
	Salinity	cry1	Overexpression of *SbCRY1a* confers oversensitive to salt stress	Zhou et al., 2018
	High-light	cry1, cry2	Cryptochromes inaffects chloroplast light-harvesting complex and redox equilibrium of photosynthetic apparatus	Walters et al., 1999; Weston et al., 2000
		cry1, cry2	Cryptochromes promotes the transcription of ROS-responsive genes	Chen et al., 2013
		cry1	Singlet oxygen–related programmed cell death is coordinated by cry1	Danon et al., 2006
		cry1, cry2	SIG5 modulates chloroplast transcripts in a cryptochromes-dependent manner	Belbin et al., 2017
		cry1	Cry1 promote the photoprotective genes expression under excessive radiation	Kleine et al., 2007; Shaikhali et al., 2012
	Heat	cry1	PIF4 interact with cry1 controlling hypocotyl elongation under high temperature	Ma et al., 2016
	Cold	–	Cryptochromes share transcription factors with cold acclimatization mechanisms	Catalá et al., 2011
	Biotic	cry1	Cry1 play a role in plant defense against pathogen attack	Zhou et al., 2017
*Brassica napus*	Drought and Salinity	cry1	Several osmotic stress responsive genes are downregulates by cry1	Sharma et al., 2014
*Solanum lycopersicum*	High-light	cry1, cry2	Cryptochromes modulates a classic high-light stress response, the anthocyanin biosynthesis	Giliberto et al., 2005; Liu et al., 2018b

## Osmotic Stress Acclimation: How Do Cryptochromes Regulate This Response?

### Drought Stress

Drought is one of the most critical factors of plant production in agricultural systems (Lesk et al., 2016; González-Villagra et al., 2017; Sseremba et al., 2018). Given the evidence regarding the mechanisms that control plant responses to drought stress (Bechtold, 2018; Hussain et al., 2018; Meng, 2018), we would not be surprised that crys are an important part of these responses (Mishra and Khurana, 2017). Previous studies have demonstrated that crys play an interesting role in Arabidopsis drought stress tolerance (Mao et al., 2005). The authors found that Arabidopsis double mutant *cry1cry2* plants were clearly more drought-tolerant than the wild type (WT) after irrigation was suspended for 1 week. However, transgenic *CRY1-ovx* plants, which overexpress the CRY1 protein, exhibited excessive water loss, which was a response mainly associated with the enhanced stomatal aperture of the transgenic plants. This response seems to be strongly related to crys, particularly through their interaction with the downstream signaling protein CONSTITUTIVELY PHOTOMORPHOGENIC 1 (COP1) (Kang et al., 2009); COP1 represses stomatal opening (Delgado et al., 2012). However, the mechanisms by which crys modulate water loss under drought stress are still very unclear because this event is dependent on many factors, including hormones, mainly abscisic acid (ABA) (Miller et al., 2010; Saradadevi et al., 2017), and interactions with other BL photoreceptors, such as phototropins (Mao et al., 2005).

Regarding ABA, some evidence shows that this hormone is part of cry signaling during water stress. For example, transgenic lines of Arabidopsis overexpressing *TaCRY1*a and *TaCRY2* from monocot wheat (*Triticum aestivum*) exhibited a lower tolerance than did WT lines to osmotic stress (300 mM mannitol) and an ABA exogenous treatment (0.3 or 10 μM ABA) throughout germination and postgermination development (Xu et al., 2009). Furthermore, these authors also showed the differential expression of *responsive to desiccation 29A* (*RD29A)* and *alcohol dehydrogenase 1* (*ADH1)*, two ABA/stress-responsive genes, in the transgenic lines. In fact, the plants overexpressing *TaCRY1a* showed a strong reduction in *RD29A* expression and a lower induction of *ADH1* under osmotic stress in comparison to plants overexpressing *TaCRY2*; the plants overexpressing *CRY1a* were also more sensitive to osmotic stress. In addition, *NAD(P)-binding Rossmann-fold superfamily protein* (*ABA2)*, which is a gene involved in adjusting ABA biosynthesis, was downregulated in *TaCRY1a-GFP* transgenic lines under ABA treatment and was downregulated in either *TaCRY1a-GFP* or *TaCRY2-GFP* transgenic lines under mannitol treatment, showing how crys coordinate molecular responses under drought via interactions with this stress hormone (Xu and Ma, 2009).

Although crys have been widely investigated in the Arabidopsis plant model (Liu et al., 2018a), recent molecular evidence in *Brassica napus*, an agriculturally important crop, has shown an interaction between cry1 and drought stress-related genes (Sharma et al., 2014). Moreover, *B. napus* cry1 overexpression (*OE-BnCRY1*) resulted in plants that were very sensitive to stress induced by a low osmotic potential (mannitol) when compared to WT, whereas the plants that were transformed with the antisense for cry1 (*AS-BnCRY1*) were more tolerant. Interestingly, cry1 overexpression reduced the transcript levels of genes related to the responses induced by water deficits (e.g., *late embryogenesis abundant protein 4-1*, *LEA4-1*; *dehydrin family protein*, *RAB18*; *nitrate reductase 1*, *NIA1*; *ascorbate peroxidase 1*, *APX1*; and *NAC domain containing protein 60*, *NAC060*). Indeed, these observations show us the involvement of crys in the drought stress acclimation responses. However, several questions, such as those related to cry stress response cascades and crosstalk mechanisms in many plant species under water deficit conditions, illuminate a new path for future research. Thus, molecular manipulation of plant crys or their signaling components, through the photoreceptor engineering, could potentially play a key role in improving drought tolerance traits.

### Salt Stress

In addition to the fact that drought and salt stress can share a common signaling pathway mainly due to their osmotic effects (Chaves et al., 2009), it was also shown that crys are part of the salt stress response via an ionic effect. Xu et al. (2009) observed that transgenic lines of Arabidopsis overexpressing *TaCRY1*a and *TaCRY2* (from *T. aestivum*) were more highly sensitive to high salt stress (120 mM NaCl) when compared with the watered control. Although salt stress impaired WT germination, this effect was more harmful to the transgenic lines than to the WT. However, the plants overexpressing *TaCRY1a* were more salt-sensitive than the plants overexpressing *TaCRY2*. In addition, real-time PCR revealed a remarkable increase in *TaCRY2* transcripts in Arabidopsis roots treated with salt stress (250 mM NaCl) after 12 h of stress induction for a 28 h period. Interestingly, the authors found that transcription of TaCRY1a was induced by salt stress after 24 h of exposure to the stressor agent.

An additional molecular event that supports the role of crys in salt stress responses comes from the overexpression of the *SbCRY1a* gene of sweet sorghum (*Sorghum bicolor*) in Arabidopsis under salinity conditions, in which a wide range of stress-responsive genes were modified (Zhou et al., 2018). Notably, the *cry1* mutant had a greater germination and seedling survival rate than the WT plants in response to ABA and salt conditions, showing more tolerance to salinity. On the other hand, the transgenic line *SbCRY1a* was oversensitive to salt stress. These mechanisms are likely regulated through the “LONG HYPOCOTYL 5-ABA INSENSITIVE 5” (HY5-ABI5) regulon, which triggers the differentiated expression of ABA/stress-responsive transcripts (*RD29A* and *RD22*), as speculated by the authors. Indeed, cry1 has been shown to control salt stress responses by ABA-dependent signaling pathways (Sharma et al., 2014). Obviously, these results allow researchers to provide more evidence on how cry signal transduction affects plant acclimation in salinity environments.

## Blue Light Mediates Light Stress Responses Through Cryptochromes

The metabolic adjustment of plants in stressful environments of excess or limited light depends on complex mechanisms that are already well established (Demmig-Adams and Adams, 1992; Demarsy et al., 2018). For instance, responsive molecules have important roles in plant photoprotection against high radiation, including anthocyanin accumulation (Petrussa et al., 2013). Notably, BL modulation of anthocyanin accumulation in Arabidopsis seedlings is cry-dependent because *cry1* C-terminal mutant alleles are known to have altered anthocyanin levels (El-Esawi et al., 2017). In addition, leaf anthocyanin accumulation increased with the overexpression of *CRY1a* in tomato (*Solanum lycopersicum* L.) (Liu et al., 2018b). These authors showed that anthocyanin accumulation was related to variations in the mRNA and protein levels of the LONG HYPOCOTYL 5 (HY5) transcription factor, which interacts with the promoters of genes for anthocyanin biosynthesis (*dihydroflavonol 4-reductase*, *chalcone synthase 1* and *2*) (Figure 1B). Similarly, it was also reported that *CRY2* overexpression (*OE-LeCRY2*) in seedlings and leaves of tomato resulted in plants with an abundant accumulation of anthocyanins (Giliberto et al., 2005), indicating the pivotal role of crys on anthocyanin accumulation, even though light stress was not directly associated with the pigments in the above works.

Nevertheless, numerous studies have highlighted the participation of crys in other high light acclimation responses, such as the regulation of redox equilibrium of photosynthetic electron transport chain under high light stress (Walters et al., 1999). Moreover, Weston et al. (2000) observed that crys modulate key photoprotective components, even the chlorophyll a/b ratio and light-harvesting complex of photosystem II (LHCB), in high white light. WT Arabidopsis and its background *cry1* and *cry2* mutants and *cry1cry2* double mutant were submitted to continuous white light under a high (600 μmol m^−2^ s^−1^) or low (60 μmol m^−2^ s^−1^) intensity. In this context, the double mutant *cry1cry2* showed a strong decrease in LHCB accumulation and in the chlorophyll a/b ratio. Currently, strong evidence shows that crys can be part of the response to a light-induced redox imbalance through their interaction with the oxidative stress system (Danon et al., 2006) and specifically through their function in ROS production (Consentino et al., 2015), including the modifications of transcript levels of *ascorbate peroxidase 2* (*APX2)*, *salt tolerance zinc finger* (*ZAT10)*, sigma factor binding protein 1 (*SIB1)*, *ethylene responsive element binding factor 4* (*ERF4)*, and *NAD(P)H dehydrogenase B2* (*NDB2)* in Arabidopsis (Chen et al., 2013; Figure 1B). More recently, it was shown that the circadian regulation of sigma factor SIG5 transcription, which is a part of the chloroplast signaling pathway in response to light stress adaptation, is predominantly dependent on BL and crys (Belbin et al., 2017). Certainly, nuclear gene expression is severely damaged when light intensities are greater than the maximum potential of the chloroplast electron capacity (Demarsy et al., 2018; Foyer, 2018). In relation to this evidence, Shaikhali et al. (2012) identified two zinc finger GATA-type transcription factors as essential regulators of the photoprotective mechanisms orchestrated by crys. Through cry1 coordination, “ZINC FINGER PROTEIN EXPRESSED IN INFLORESCENCE MERISTEM LIKE1” (ZML1) and “ZML2” play a fundamental role in ROS scavenger responses to excess light. In another way, some discussions have been considered about ROS biosynthesis during crys inactivation (Consentino et al., 2015; Jourdan et al., 2015; El-Esawi et al., 2017). However, the link between the ROS produced under light and dark conditions merits further investigation (Figure 1B).

Regarding specific short-wavelength light signaling, such as UV-B, it has also been speculated that crys are part of these responses (Fortunato et al., 2015). In general, it is well known that UV-B light causes photodamage to DNA polymers and affects the electron balance in photosystem II (Czégény et al., 2016; Dotto and Casati, 2017), and it has been shown that cry-DASH homologs participate in restoring the photosynthetic efficiency of the photosystem II reaction center complexes in photosynthetic organisms (Vass et al., 2014). However, it is still hard to understand the mechanisms in which crys are part of high light stress response because a large system of photoprotective mechanisms is triggered under this condition, besides the fact that BL induces UV-B stress tolerance (Adamse et al., 1994; Hoffmann et al., 2015), and numerous high light-responsive genes are modulated by cry1 in Arabidopsis (Kleine et al., 2007). Therefore, there are potential opportunities for future studies about how cry signal transduction is involved in light stress responses to ambient fluctuations.

## What Can Cryptochromes Reveal About Other Plant Stresses?

### Temperature Stress

Temperature stress triggers a large amount of metabolic damage in plants with well-known effects on protein stability and enzymatic reactions (Szymańska et al., 2017). In addition, the changes between the regulatory mechanisms of ROS and multiple pathways under heat stress have been recently reviewed, showing that downstream heat shock protein (HSP) is largely associated with these responses (Chen et al., 2018; Suzuki and Katano, 2018). In view of this, the HSP transcription profile is strongly regulated by crys (Facella et al., 2008; Yang et al., 2008), allowing a new perspectives on this BL photoreceptor in high temperature responses. A previous study demonstrated that cry1 repressed auxin biosynthesis to acclimate to heat stress, resulting in morphological changes in Arabidopsis seedlings (Ma et al., 2016). In addition, cry1 interacted with promoters such as *flavin-binding monooxygenase family protein* (*YUC8)*, *indole-3-acetic acid inducible 19* (*IAA19)*, and *indole-3-acetic acid inducible 29* (*IAA29)* under high temperature in a PHYTOCHROME-INTERACTING FACTOR 4 (PIF4)-dependent manner, which is an important part of BL-dependent signal transduction. On the other hand, the mechanisms of plant acclimation to cold stress in Arabidopsis include HY5, COP1, and Z-box, which are involved in these responses (Catalá et al., 2011); Z-box is a regulatory *cis*-element located in the promoter of responsive genes, such as HY5 transcription factor (Yadav et al., 2005). Coincidentally, these key regulators of light signaling are known to be mediated by crys through various interactions with other photoreceptors and signaling molecules (Mishra and Khurana, 2017), causing speculation that these BL photoreceptors have a role in a part of low temperature tolerance. However, as can be observed in this topic, temperature response modulated by crys in plants is still uncommon, but intensive research on this subject should be considered.

## Epilogue

The role of crys in several events related to plant growth and development has been well established, including biotic stress response (Jeong et al., 2010; Wu and Yang, 2010; Zhou et al., 2017). However, few papers consider the participation of these photoreceptors in one of the most scientifically explored topics, abiotic stress in plants. Although much still needs to be revealed and understood about the subject, this review aimed to address and discuss different insights in the key function of BL in the acclimation to abiotic stresses via cry regulation. Thus, in this paper, we focus on compiling the responses to abiotic stress mediated by crys, although biotic stress is an interesting matter that may need to be intensively addressed (Table 1). In fact, since large changes in agronomically desirable traits have been shown in mutants and transgenic lines of important crops (Mawphlang and Kharshiing, 2017), regarding stress tolerance, potential future perspectives could be generated for agriculture on the manipulation of this multifaceted photoreceptor. However, we are aware that the subject is complex because it involves the interaction of crys with other photoreceptors as well as with the signaling of other important stress-modulating molecules (Figure 1B), such as plant hormones (Casal, 2000; Warpeha and Montgomery, 2016; Xu et al., 2018). Therefore, we encourage new investigations that clarify the underlying mechanisms of cry-related stress responses in different plant species. Certainly, these efforts will contribute to revealing other crys functions in the acclimation to stressful environmental conditions. Overall, exploring the molecular mechanisms by which crys mediate abiotic stress responses are issues that deserve further investigation.

## Author Contributions

VD-D and RC designed this review, wrote the manuscript, and approved it for publication.

## Conflict of Interest Statement

The authors declare that the research was conducted in the absence of any commercial or financial relationships that could be construed as a potential conflict of interest.
